# Pulmonary adenocarcinoma with massive lymphocytic infiltration: a case report with review of the literature of a rare histological entity with a peculiar biological behaviour

**DOI:** 10.1186/1471-2466-13-44

**Published:** 2013-07-11

**Authors:** Alessandro Del Gobbo, Stefano Fiori, Gabriella Gaudioso, Mario Nosotti, Guido Coggi, Silvano Bosari, Stefano Ferrero

**Affiliations:** 1 Division of Pathology, Fondazione IRCCS "Ca' Granda" - Ospedale Maggiore Policlinico, University of Milan Medical School, Via Francesco Sforza, 35, Milano, 20100, Italy; 2 Division of Thoracic Surgery, Fondazione IRCCS "Ca' Granda" - Ospedale Maggiore Policlinico, University of Milan Medical School, Milan, Italy; 3 Department of Clinical/Surgical Pathophysiology and Organ Transplant, University of Milan Medical School, Milan, Italy; 4 Department of Biomedical, Surgical and Dental Sciences, University of Milan Medical School, Milan, Italy

**Keywords:** Pulmonary adenocarcinoma, Tumoral host response, Lymphocytic infiltration

## Abstract

**Background:**

Tumors with a massive inflammatory infiltration are described in several organs. There is agreement about considering the inflammatory infiltration as the host’s immune response to neoplastic cells; such neoplasms indeed have a better prognostic outcome than non-inflammatory counterparts. Only seventeen cases of pulmonary adenocarcinoma with massive lymphocytic infiltration (AMLI) have been reported in literature so far.

**Case presentation:**

We present a case of pulmonary adenocarcinoma with massive lymphocytic infiltration occurring in a 71 years old male smoker. He came under our attention because of dyspnea, and underwent a left lower lobectomy. Histological examination showed a moderately differentiated (G2) acinar adenocarcinoma associated with a stromal desmoplastic reaction and a massive inflammatory infiltration, made up mostly of CD3+ lymphocytes. pTNM stage was pT2a, N0 (clinical stage: Ib).

Molecular testing of EGFR gene showed no mutations and immunohistochemistry for ALK resulted negative.

EBV infection was ruled out by EBV in situ hybridization.

**Conclusions:**

Literature review showed seventeen similar cases, with a 16/1 male/female ratio and a mean age of 70,2 years. In eight out of seventeen cases EBV-infection was demonstrated with immunohistochemical or molecular biology techniques.

Similarly to the cases previously reported in literature our patient is a male smoker, without lymph node metastasis and he is still alive after a follow-up period of six months without recurrent or residual disease.

Because of histological, biological and clinical peculiarity, we propose to take into account pulmonary adenocarcinomas with massive inflammatory infiltration for a separate pathological classification.

## Background

Tumors with a massive inflammatory infiltration are found in different sites such as gastrointestinal tract, liver, breast and female genital system [1-6].

The inflammatory infiltrate, which is thought to depict the immune response to neoplastic cells, has been studied mainly in large bowel cancer, and is linked to an improved prognostic outcome. It is typically mixed and made up of lymphocytes, macrophages, dendritic cells and granulocytes [7].

Lung pathology displays a well-defined tumor entity with massive lymphoid infiltration, lymphoepithelioma-like carcinoma (LELC). It is a subtype of large cell carcinoma often related, especially in Asiatic patients, to Epstein-Barr Virus (EBV) infection.

This entity shares most pathological features with the nasopharingeal counterpart [8].

Pulmonary adenocarcinoma with massive lymphocytic infiltration (AMLI) is not included as a separate entity in current World Health Organization (WHO) classification of tumors or in the new multidisciplinary pathological classification published in 2011 by Travis et al. [9]; though, it is recognized by some sources as a rare histological variant of pulmonary adenocarcinoma [10].

To the best of our knowledge, only seventeen cases of AMLI are reported in literature so far.

AMLI is distinguished from ''classic'' acinar adenocarcinoma by the tendency to occur in advanced-age males; prognosis is way better since the massive flogistic infiltrate reflects the active host's immune response. Moreover, cytological diagnosis may be misleading, because of the relative paucity of neoplastic cells and the wide predominance of inflammatory subsets [11].

Glandular acinar differentiation was found in all cases; a marked fibrous stroma was a common finding too. Typically, inflammatory cells are more represented and tend to overwhelm the neoplastic counterpart, with diffuse infiltration and effacement of tumoral glands. This, as said before, leads to a relative paucity of neoplastic cells [12,13].

In nine cases [11,12], EBV infection was ruled out by immunohistochemical or molecular biology techniques, and in the other eight cases reported in literature [14] EBV positivity was demonstrated in neoplastic tissue.

Further immunophenotypical characterization of the infiltrate showed a prevalent T-lymphoid distribution, with a greater extent of CD8+ cells. B-cells, plasma cells, macrophages and granulocytes were represented at a lower amount; prominent reactive germinal centers were found in a minority of cases [12,13].

We report a case of a 71 years old man who underwent a lower left lobectomy, with a postoperative histopathological diagnosis of pulmonary acinar adenocarcinoma with massive lymphocytic infiltration.

## Case presentation

A 71 years old male came to our attention because of dyspnea during the last three months.

A chest computed tomography scan showed a 35 × 25 mm central pulmonary nodule of the lower left lobe with no evidence of thoracic lymphoadenopaties.

A positron emission tomography (PET) scan demonstrated the concentration of fluorodeoxyglucose (FDG) in that nodule, with a peripheral rim of hyperfixation (standard uptake value max 6.9).

The patient underwent a left lower lobectomy with omolateral mediastinal lymphoadenectomy; gross examination of the specimen showed a 3.8 cm greyish nodule, with stellate margins and no involvement of the visceral pleura.

**Figure 1:**
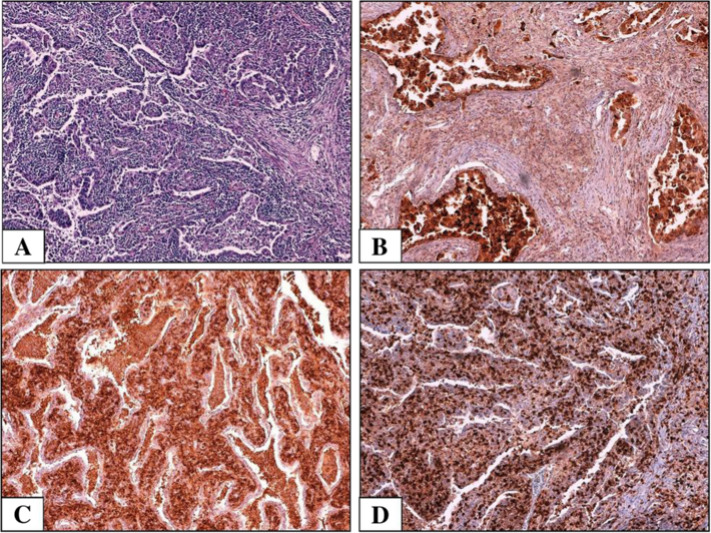
**Histological and immunoistochemical features of the case. A**: Neoplastic glands surrounded and infiltrated by a massive inflammatory infiltration (H&E, 10×); **B**: Immunoreactivity for Napsin-A confirms the glandular nature of the tumor (10×); **C**,**D**: The infiltrate was composed predominantly of CD8+ lymphocytes **(C**, 10×**)** and CD4+ lymphocytes **(D**, 10×**)**.

Light microscopy showed a moderately-differentiated (G2) acinar adenocarcinoma, with marked stromal fibrosis. Necrosis was not found (Figure 1A).

A massive lymphocytic reaction, with a diffuse pattern of growth and several aspects of infiltration of neoplastic glands was present in the tumoral stroma. No germinal centers were highlighted. Immunohistochemistry revealed a strong positivity of the tumoral cells for TTF-1 (DAKO, clone 86763/1) and Napsin-A (DAKO, policlonal) antibodies, confirming the glandular histogenesis and the pulmonary primitivity (Figure 1B).

The lymphoid component was found to be policlonal and made up mainly by CD3+ (DAKO, clone 2GV6, Rabbit) T-cells. Cytotoxic (CD8+, DAKO, clone C8/144B) T-lymphocytes were more represented than helper (CD4+, DAKO, clone 5835) cells (Figure 1C and 1D). EBV infection was excluded by an EBV Encoded Small RNAs (EBER) probe negative in situ hybridization.

According to these histological findings, we made a diagnosis of pulmonary adenocarcinoma with massive lymphocytic infiltration.

All the mediastinal lymphnodes showed antracosis and sinus hystiocitosis without evidence of metastasis.

Molecular testing of the EGFR gene (regions investigated: exon 18 codon 719, exon 19 codon 746–750, exon 21 codons 858 and 861) performed by pyrosequencing, using the "Pyromark Q96 ID System" with the diagnostic kit IVD / CE EGFR TKI response (Diatech Pharmacogenetics srl) showed no mutations.

Molecular testing of the KRAS gene (regions investigated: codon 12, 13, 61 and 146) performed by pyrosequencing using the "Pyromark Q96 ID System" with the diagnostic kit IVD / CE Anti-EGFR mAb response (KRAS status) [Diatech Pharmacogenetics Srl] showed a wild type phenotype.

Lastly, immunostaining for ALK (Novocastra, clone 5A4) resulted negative.

After a follow up period of six months, the patient is in good health with no evidence of residual or recurrent disease.

## Conclusions

An inflammatory response of a variable degree, interpreted as the host's attempt to contain neoplastic growth, is detectable in most tumors [7]. Though, a relevant inflammatory infiltration can be found only in small percentages of tumors.

The current (2004) WHO classification of tumors of the Lung, Pleura, Thymus and Heart contains an only entity associated with intratumoral inflammatory infiltrate: lymphoepithelioma-like carcinoma, which is classified as a histological variant of large cell carcinoma. This neoplasm is overall rare but accounts for around 1% of all the lung tumors in China.

It is made up by poorly differentiated epithelial cells with a prominent lymphoid stroma, and is strictly associated with EBV infection, detectable in nearly 100% of cases [8].

In our case, the neoplastic component is represented by acinar glands, with a smaller account of solid areas. In support of the glandular nature of this neoplasm there is immunohistochemical positivity for Napsin-A and TTF-1 antibodies.

Literature displays, after an extensive search, only three other studies concerning adenocarcinoma with massive lymphocytic infiltration (AMLI).

Minami et al. described three cases of AMLI. All data, apart from the prevalent CD4+ phenotype of stromal lymphocytes, overlap our findings [12].

Tsuta et al. reported six cases of AMLI; in this case report, the prevalent lymphocyte phenotype was CD8+. All nine cases showed a diffuse pattern of growth of the lymphoid infiltrate, with only few germinal centers and a smaller amount of peripheral distributed B-cells [13]. In these cases, EBV infection was ruled out.

Gomez-Roman et al. reported eight cases of lung adenocarcinoma with a diffuse lymphoid stroma and a tubuloglandular pattern, associated with EBV infection [14].

The most relevant findings of these three papers are reported in Table 1.

**Table 1:** Cases of pulmonary adenocarcinomas with massive lymphocytic infiltration reported in literature

**Case N.**	**Age**	**Sex**	**Smoke**	**Stage**	**Follow-up (mths)**	**EBV infection**
1	68	M	n.a.	IIIb	Alive (62)	Negative
2	75	M	n.a.	IIIa	Dead (26)	Negative
3*	64	M	n.a.	Ia	Alive (98)	Negative
4	70	F	+	IIIa	Alive (98)	Negative
5	55	M	+	IIIa	Alive (67)	Negative
6	85	M	+	n.a.	Alive (57)	Negative
7	72	M	+	IIa	Alive (48)	Negative
8	68	M	+	Ia	Alive (17)	Negative
9**	75	M	+	IIIa	Alive (12)	Negative
10	72	M	n.a.	Ib	Dead (26)	Positive
11	61	M	n.a.	Ia	Alive (40)	Positive
12	54	M	n.a.	Ia	Lost follow-up	Positive
13	70	M	n.a.	Ib	Lost follow-up	Positive
14	69	M	n.a.	Ib	Lost follow-up	Positive
15	55	M	n.a.	IIIa	Dead (1)	Positive
16	67	M	n.a.	IIIa	Alive (12)	Positive
17***	58	M	n.a.	Ia	Alive (36)	Positive
18****	71	M	+	Ib	Alive (6)	Negative

(*Case 1 through 3: Minami Y et al., Lung Cancer, 2003; **Case 4 through 9: Tsuta K et al., Am J Clin Path, 2005; ***Case 10 through 17: Gomez-Roman et al., Modern Pathology, 2009; ****Case 18: current report).

In our opinion, the main data to focus on is that AMLI more frequently occurs in advanced-age male with a significant better prognostic outcome than classic acinar adenocarcinoma despite the presence of lymph node metastases.

In our case, the tumor is characterized by the typical molecular profile of lung carcinomas arising in smokers, with EGFR and ALK wild type phenotype [15,16].

Although lacking a molecular target for targeted therapy and expecting a poor outcome, these tumors have a good prognosis, due to their biology and to the host immune defense.

Peculiar histological, molecular and clinical features may suggest a distinct classification of the tumor. Unfortunately, prevailing data are not adequate to assess real incidence of AMLI. It would be advantageous to furtherly consider AMLI in future studies, in order to be able to place it in a separate pathologic category.

### Consent

Written informed consent was obtained from the patient for publication of this case report and any accompanying images. A copy of the written consent is available for review by the Editor of this journal.

## Competing interests

The authors declare that they have no competing interests.

## Authors’ contributions

ADG and SF analyzed, interpreted the patient’s data and drafted the manuscript. GG performed immunohistochemical and molecular analysis. MN revised the clinical data; SF, GC and SB revised the pathology data and supervised the case report. All authors read and approved the final manuscript.

## Pre-publication history

The pre-publication history for this paper can be accessed here:

http://www.biomedcentral.com/1471-2466/13/44/prepub
